# AAV GCG-EGFP, a new tool to identify glucagon-secreting α-cells

**DOI:** 10.1038/s41598-019-46735-2

**Published:** 2019-07-25

**Authors:** Eva Tudurí, Maria M. Glavas, Ali Asadi, Robert K. Baker, Cara E. Ellis, Galina Soukhatcheva, Marjolaine Philit, Frank K. Huynh, James D. Johnson, C. Bruce Verchere, Timothy J. Kieffer

**Affiliations:** 1 Department of Cellular and Physiological Sciences, Life Sciences Institute, University of British Columbia, Vancouver, British Columbia Canada; 2 Centro de Investigación Biomédica en Red de Diabetes y Enfermedades Metabólicas Asociadas (CIBERDEM), Madrid, Spain; 3 Instituto de Investigación, Desarrollo e innovación en Biotecnología Sanitaria de Elche (IDiBE), Elche, Spain; 4 Department of Surgery, University of British Columbia, Vancouver, British Columbia Canada; 5 Department of Pathology and Laboratory Medicine, BC Children’s Hospital Research Institute, Vancouver, British Columbia Canada; 6 Department of Biological Sciences, San Jose State University, San Jose, CA USA

**Keywords:** Genetic vectors, Diabetes

## Abstract

The study of primary glucagon-secreting α-cells is hampered by their low abundance and scattered distribution in rodent pancreatic islets. We have designed a double-stranded adeno-associated virus containing a rat proglucagon promoter (700 bp) driving enhanced green fluorescent protein (AAV GCG-EGFP), to specifically identify α-cells. The administration of AAV GCG-EGFP by intraperitoneal or intraductal injection led to EGFP expression selectively in the α-cell population. AAV GCG-EGFP delivery to mice followed by islet isolation, dispersion and separation by FACS for EGFP resulted in an 86% pure population of α-cells. Furthermore, the administration of AAV GCG-EGFP at various doses to adult wild type mice did not significantly alter body weight, blood glucose, plasma insulin or glucagon levels, glucose tolerance or arginine tolerance. *In vitro* experiments in transgene positive α-cells demonstrated that EGFP expression did not alter the intracellular Ca^2+^ pattern in response to glucose or adrenaline. This approach may be useful for studying purified primary α-cells and for the *in vivo* delivery of other genes selectively to α-cells to further probe their function or to manipulate them for therapeutic purposes.

## Introduction

Glucagon-secreting α-cells play a fundamental role in regulating glucose homeostasis. While insulin is released from β-cells after meal ingestion in order to stimulate glucose uptake^1,2^, glucagon is secreted from α-cells in conditions of fuel demand to induce hepatic glucose production^3,4^. Hyperglucagonemia, a state that worsens hyperglycemia, has been observed in humans with diabetes^5^ and in animal models of diabetes^6–10^. Importantly, glucagon action appears to be necessary for hyperglycemia to occur in diabetes^11,12^. However, there is a considerable lack of information about the mechanisms that govern glucagon secretion. This is largely a consequence of the shortage of methods to identify and/or isolate α-cells.

A common strategy to identify glucagon-secreting α-cells *in vivo* consists of crossing mice expressing Cre recombinase under the glucagon promoter (Gcg-Cre mice) with reporter mice containing a loxP site transcriptional STOP sequence upstream of the open reading frame of a fluorescent protein^13–16^. Although these double transgenic models allow for rapid visualization of islet α-cells, limitations arise when studies require the use of other mouse strains. Therefore, an approach that permits acute expression of fluorescent proteins in α-cells, independent of the rodent strain, would be ideal for a better understanding of the physiology of this cell population.

The adeno-associated viruses (AAVs) are currently one of the preferred vectors to deliver transgenes. Among their features it is worth highlighting their minimal immunogenicity, their ability to infect both dividing and non-dividing cells, and the resulting long term transgene expression^17–19^. Due to these characteristics, AAVs have been widely employed in clinical reports^20^. Several reports performed in animal models have shown good infection of pancreatic cells by means of AAV6, AAV8 or AAV9^21–25^, although, to our knowledge, only a few studies have achieved transduction of α-cells by *in vivo* delivery of AAVs^15,23,25^. In those reports the authors did not use an α-cell specific promoter, therefore transduction included a large fraction of other pancreatic cells including β-cells and acinar cells. The aim of our study was to specifically target α-cells by means of a viral vector. We therefore designed a double stranded AAV8 carrying the enhanced green fluorescent protein (EGFP) transgene under a 700 bp fragment of the rat glucagon promoter (AAV GCG-EGFP). Here we show that delivery of this AAV GCG-EGFP, by either the intraperitoneal or intraductal route, allows for specific expression of EGFP in α-cells without affecting cell function. Our results suggest that AAVs may provide an effective means for gene therapy approaches targeting α-cells.

## Results

### AAV GCG-EGFP administration leads to specific EGFP expression in pancreatic α-cells

**Figure 1:**
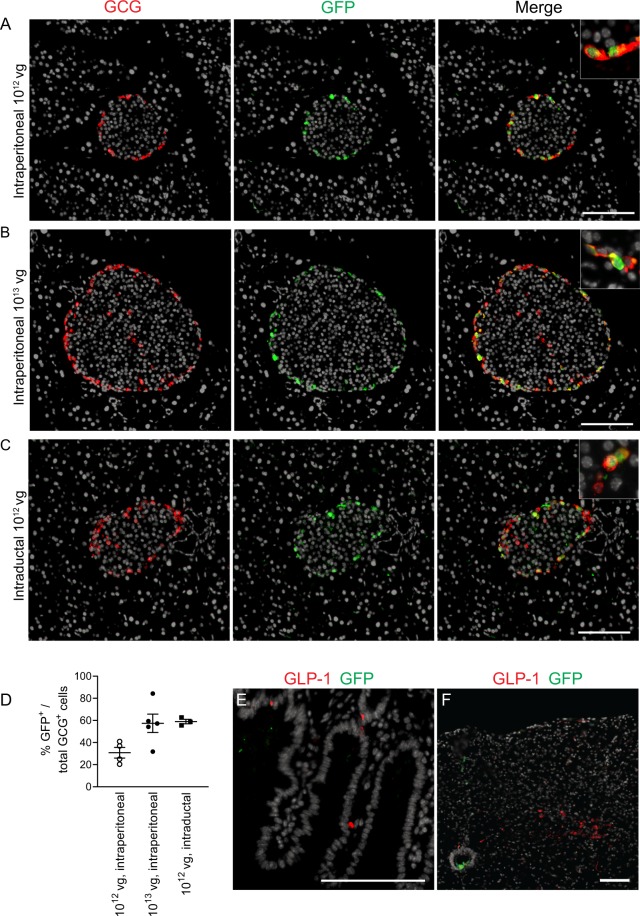
AAV GCG-EGFP leads to α-cell EGFP expression. Pancreas sections from adult C57BL/6 mice treated with AAV GCG-EGFP by (**A**) single intraperitoneal injection of 10^12^ vg, (**B**) single intraperitoneal injection of 10^13^ vg, and (**C**) single intraductal injection of 10^12^ vg. Glucagon (red), GFP (green), and DAPI (grey). (**D**) Quantification of cells with colocalization of both GFP and GCG as a ratio of total GCG-positive cells, from pancreas sections. (**E**) Small intestine section and (**F**) brainstem section at the level of the solitary tract nucleus, stained for GLP-1 (red), GFP (green), and DAPI (grey), from a mouse treated with 10^13^ vg of AAV GCG-EGFP by single intraperitoneal injection. Non-specific GFP staining can be seen in the lumen of the central canal in brainstem sections. Scale bars = 100 μm. AAV: adeno-associated virus; GCG: glucagon; GFP: green fluorescent protein; GLP-1: glucagon-like peptide 1.

To examine the islet distribution of EGFP expression after administration of AAV GCG-EGFP, adult C57BL/6 mice were treated with different doses of the AAV by a single intraperitoneal injection and their pancreata were harvested 5 months later. The immunohistochemical analysis of pancreas sections revealed specific EGFP expression in the α-cell population within the islets after administration of 10^12^ and 10^13^ viral genomes (vg) of AAV GCG-EGFP (Fig. 1A,B), whereas no GFP staining was observed in pancreas from mice treated with 10^10^ or 10^11^ vg of the AAV (Supplementary Fig. S1). In a parallel study, AAV GCG-EGFP was delivered by intraductal injection at a dose of 10^12^ vg. This route of administration allows for the direct delivery of the vector to the pancreas, therefore reducing the infection of other tissues and increasing the viral load to pancreatic cells^23^. Two months after AAV GCG-EGFP intraductal delivery, pancreata were removed and fixed for immunofluorescence analysis, which confirmed specific staining of GFP in pancreatic glucagon positive (GCG^+^) cells (Fig. 1C). Quantification of pancreas section immunostaining (Fig. 1D) indicated that 30.8 ± 9.7% and 57.4 ± 8.3% of GCG^+^ cells were also immunoreactive for GFP after intraperitoneal administration of 10^12^ and 10^13^ vg of AAV GCG-EGFP, respectively, and 59.0 ± 2.0% after intraductal administration of 10^12^ vg of AAV GCG-EGFP. Only rare GFP^+^ cells that did not colocalize with GCG were observed in islets (frequency of ~0.1 cells/islet).

Glucagon promoter activity is present not only in pancreatic α-cells^26^, but also in intestinal L-cells^27^ and in a small population of neurons in the brainstem^28^. Hence, we also performed immunohistochemistry of small intestine (Fig. 1E), brainstem (Fig. 1F) and liver (Supplementary Fig. S2) sections after intraperitoneal administration of the highest dose tested (10^13^ vg) and no GFP staining was observed.

### AAV GCG-EGFP as a tool to obtain an enriched fraction of α-cells

**Figure 2:**
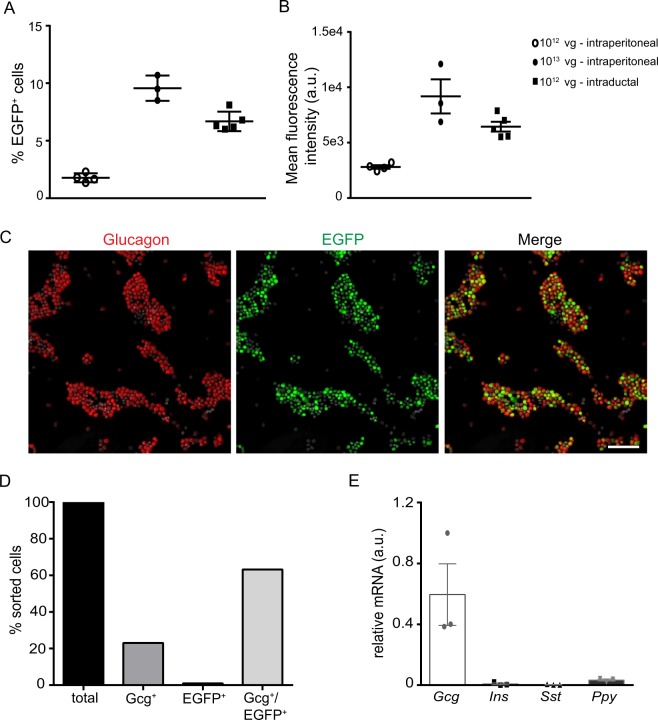
Administration of AAV GCG-EGFP allows for cell sorting of α-cells. (**A**) Percentage of total islet cells and (**B**) mean fluorescence intensity of EGFP^+^ cells, sorted based on EGFP fluorescence, after either intraperitoneal administration of 10^12^ (n = 4) and 10^13^ vg (n = 3) or intraductal administration of 10^12^ vg (n = 5) of AAV GCG-EGFP. (**C**) Immunocytochemistry for glucagon (red), GFP (green) and DAPI (grey) in a population of islet cells sorted after intraperitoneal administration of 10^12^ vg AAV GCG-EGFP and (**D**) percentage of glucagon positive GFP negative (GCG^+^), glucagon negative GFP positive (GFP^+^), and both glucagon and GFP positive (GCG^+^/GFP^+^) cells based on immunohistochemistry of sorted cells. (**E**) mRNA levels of islet hormones in the EGFP positive fraction of sorted cells (n = 3). AAV: adeno-associated virus; EGFP: enhanced green fluorescent protein; vg: viral genomes; GCG: glucagon; Ins: insulin; Sst: somatostatin; Ppy: pancreatic polypeptide. Data are expressed as mean ± SEM.

Since AAV GCG-EGFP leads to specific EGFP expression in α-cells, we investigated whether the endogenous EGFP fluorescence intensity resulting from the transgene expression could be sufficient to sort a purified population of α-cells from a mixed population of pancreatic endocrine cells. Two to 3 months after mice had been treated with AAV GCG-EGFP, by intraperitoneal or intraductal injection, pancreatic islets were isolated and dispersed into single cells for FACS based on EGFP fluorescence. The results obtained indicated that 1.8 ± 0.2% and 9.6 ± 0.6% of the total islet cells sampled were sorted as EGFP^+^ cells after intraperitoneal administration of 10^12^ and 10^13^ vg of AAV GCG-EGFP, respectively, and 6.7 ± 0.4% after intraductal administration of 10^12^ vg of AAV GCG-EGFP (Fig. 2A). The intensity of the signal was also analyzed and found to be three-fold higher in the 10^13^ vg intraperitoneal group compared to the 10^12^ vg intraperitoneal group (Fig. 2B). Therefore, a dose of 10^13^ vg could be advantageous for experiments requiring a strong fluorescent signal. We next performed immunocytochemistry in the sorted cell fraction (Fig. 2C) and observed that 63% were GCG^+^/GFP^+^ cells (Fig. 2D). Approximately 23% of the cells were GFP^−^/GCG^+^. Since cells were sorted for EGFP, the detection by immunocytochemistry of GCG^+^ cells that lack GFP immunoreactivity may reflect the poorer sensitivity of immunocytochemistry versus FACS, or loss of the EGFP following FACS for instance after membrane permeabilization. It is also possible that α-cells with low GCG were not detected by immunocytochemistry, accounting for non-stained cells. Only 1% of sorted cells were GCG^−^/GFP^+^ (Fig. 2D). Thus, cell sorting based on EGFP fluorescence resulted in a population that was at least 86% GCG^+^, as determined by immunocytochemistry. These results are in accordance with the gene expression analysis of the major islet hormones transcripts (Fig. 2E), which revealed that glucagon was highly expressed in EGFP^+^ cell fractions and that some of the sorted cells expressed pancreatic polypeptide.

### AAV administration does not alter body weight, blood glucose, plasma glucagon and insulin levels, or glucose and arginine tolerance

**Figure 3:**
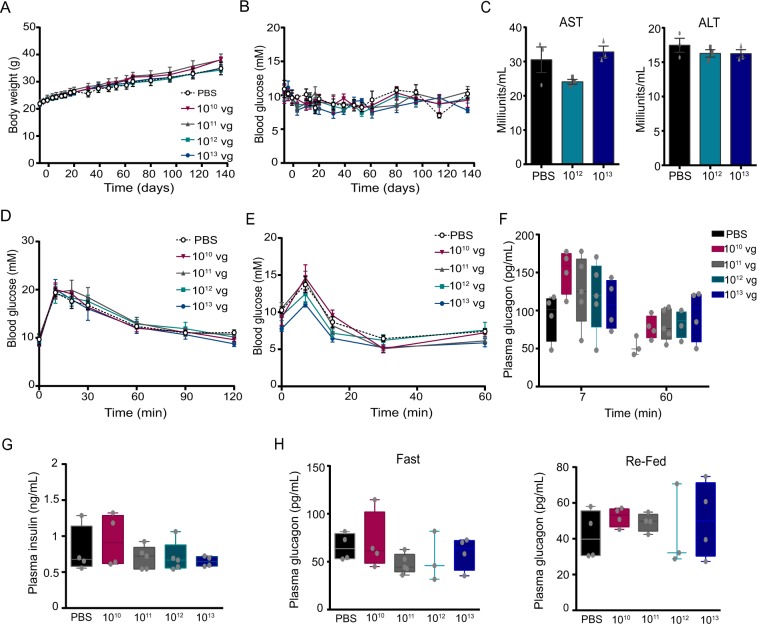
Administration of AAV GCG-EGFP does not alter body weight or glucose homeostasis. Four hour fasted (**A**) body weight and (**B**) blood glucose, before and after intraperitoneal administration of several doses of AAV GCG-EGFP from 10^10^ to 10^13^ vg on day 0. (**C**) Serum AST and ALT levels 2 weeks post intraperitoneal injection of AAV. (**D**) OGTT was performed following a dextrose load of 1.5 g/kg body weight after a 4 hour fast, 10 weeks after AAV administration. An arginine tolerance test was performed via an arginine injection of 2 g/kg body weight after a 4 hour fast, 13 weeks after AAV injection, with (**E**) blood glucose and (**F**) plasma glucagon levels measured at different timepoints following arginine administration. (**G**) Plasma insulin levels after overnight fasting and (**H**) plasma glucagon levels after overnight fasting and in the re-fed state (2 hr ad libitum feeding) 23 weeks after AAV injection. AST: aspartate aminotransferase; ALT: alanine aminotransferase; vg: viral genomes. Data are expressed as mean ± SEM. Statistical analysis was performed using Kruskal-Wallis Test or two-way ANOVA (no significant differences observed among groups). A, B, D and E, n = 4 in PBS, 10^10^ and 10^13^ groups, and n = 5 in 10^11^ and 10^12^ groups.

To determine whether the administration of AAV GCG-EGFP could alter the function of the islets of Langerhans, and consequently affect glucose homeostasis, we performed a series of *in vivo* experiments in several groups of mice treated with either different doses of AAV GCG-EGFP (10^10^, 10^11^, 10^12^ or 10^13^ vg/mouse) or PBS (control group) by a single intraperitoneal injection. Firstly, four hour fasting body weight and blood glucose levels were monitored weekly through the duration of the study (from a few days before the AAV injection until 4.5 months post-injection), and no differences were observed between the AAV-treated animals and the control group (Fig. 3A,B). Two weeks post virus administration, we ruled out possible liver damage by measuring the serum levels of aspartate aminotransferase (AST) and alanine aminotransferase (ALT) (Fig. 3C), which were not altered by AAV treatment. From 2.5 months post AAV-injections, several *in vivo* procedures were performed. An oral glucose tolerance test indicated that none of the doses tested modified glucose responses (Fig. 3D). Since glucagon secretion is stimulated by arginine^29^, we carried out an arginine tolerance test to determine whether or not the expression of EGFP in α-cells could alter their secretory capacity in response to the amino acid. All AAV-treated mice had a similar blood glucose curve in response to arginine, and glucagon secretion at 7 and 60 min post arginine did not differ among groups (Fig. 3E,F). Plasma insulin levels were analyzed following an overnight fasting and no significant differences were observed among the groups (Fig. 3G). In addition, plasma glucagon levels were measured after an overnight fast and in the re-fed state (Fig. 3H) and no differences were observed among treatments. Collectively, these results indicate that the administration of AAV GCG-EGFP had no major effect on glucose homeostasis.

### Isolated primary EGFP positive and EGFP negative α-cells display similar intracellular Ca^2+^ oscillations

**Figure 4:**
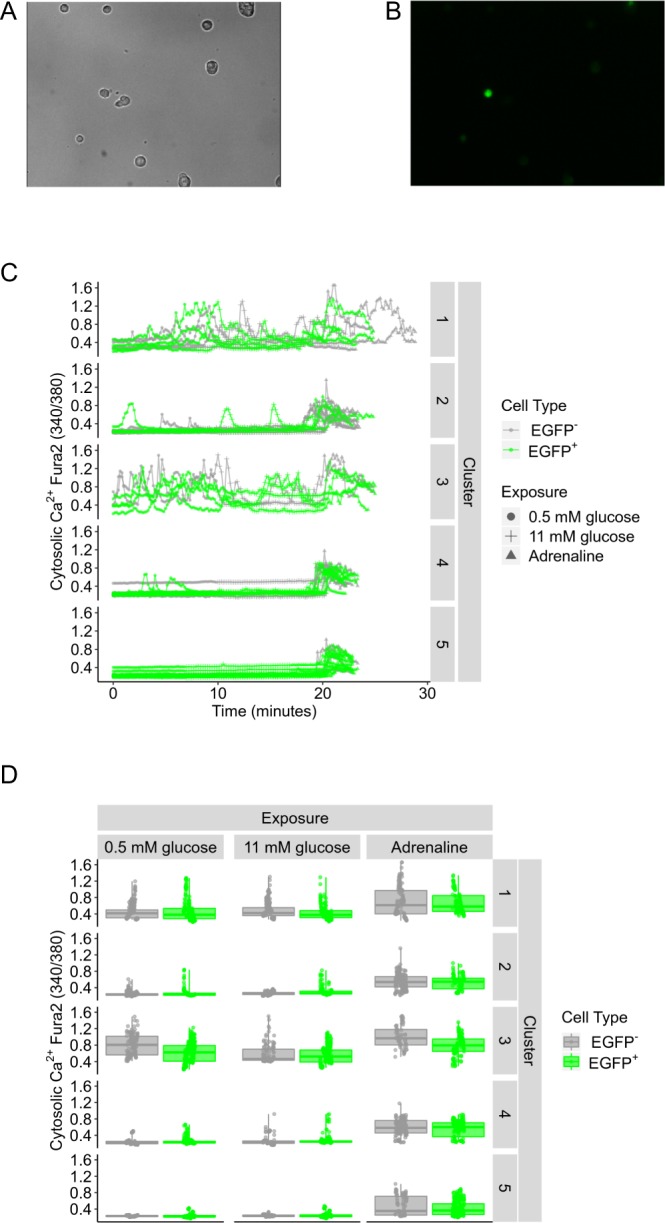
Intracellular Ca^2+^ pattern in EGFP positive and EGFP negative α-cells in response to glucose and adrenaline. (**A**) Bright field and (**B**) fluorescent EGFP signals in dispersed islet cells. (**C**) Raw Ca^2+^ traces from single EGFP^+^ (green) and EGFP^−^ (grey) α-cells in response to low glucose (0.5 mM), high glucose (11 mM) and adrenaline (5 µM). (**D**) Summary statistics for the cell types sorted by cluster. A total of 33 EGFP^+^ and 27 EGFP^−^ α-cells from 5 AAV-treated mice were imaged.

The function of EGFP-expressing α-cells was studied at the intracellular Ca^2+^ level. One month after intraperitoneal administration of 10^12^ vg of AAV GCG-EGFP, pancreatic islets were isolated and dispersed into single cells for Ca^2+^ imaging by means of the fluorescent Ca^2+^ indicator Fura 2-AM. Dispersed islet cells (Fig. 4A and B) were exposed to low (0.5 mM) and high (11 mM) glucose concentrations, as well as adrenaline (5 μM). Endogenous fluorescence (Fig. 4B) allowed for the easy identification of EGFP^+^ α-cells, and EGFP^−^ α-cells were identified by their responsivity to adrenaline. Ca^2+^ traces of 33 EGFP^+^ and 27 EGFP^−^ α-cells were imaged and studied by employing an unbiased cluster-based analysis based on trace features^30^. Bayesian Information Criterion of Gaussian mixture models of x parameters suggested 5 feature clusters in the 60 traces, with cell type not contributing to cluster identity (p = 0.09, p ≥ 0.5). EGFP^+^ and EGFP^−^ α-cells within the same cluster displayed similar responses to the different stimuli (Fig. 4C,D and Supplementary Fig. S3). These outcomes indicate that the transgene expression did not interfere with the normal intracellular Ca^2+^ pattern of the α-cell population.

## Discussion

Here we provide evidence that AAV8 is capable of directing the expression of a transgene in pancreatic α-cells following *in vivo* delivery. Among the different existing AAV serotypes, AAV6, AAV8, and AAV9  have been shown to induce transgene expression in the pancreas^15,21–23,25^; however, transduction of α-cells has thus far been difficult to achieve by means of AAVs without also transfecting a large portion of other pancreatic cell types^15,23,25^. To our knowledge, the AAV GCG-EGFP is the first successful viral vector reported to transduce a substantial number of α-cells within the pancreas with a minimum level of transduction in other pancreatic cell populations. Driven by a 700 bp fragment of the rat glucagon promoter, the EGFP transgene was expressed for at least 5 months, which is in accordance with other studies that reported long-term transgene expression in pancreatic cells by means of AAVs^22,25^.

With our AAV GCG-EGFP, the immunocytochemical analysis of the sorted EGFP^+^ fraction indicated that 86% of sorted cells expressed glucagon. Considering we sorted 86% of glucagon-secreting α-cells in the EGFP^+^ fraction, and given that mouse islets consist of 15–20% α-cells^31,32^, we estimated that intraperitoneal administration of 10^12^ or 10^13^ vg of AAV GCG-EGFP resulted in transduction of 8–10% or 41–55% of α-cells, respectively. Intraductal administration resulted in a higher rate of transduction at a dose of 10^12^ vg, with an estimated 29–38% of α-cells targeted. Indeed, the immunohistochemical analysis of individual pancreas sections showed 30.8 ± 9.7% and 57.4 ± 8.3% of α-cells immunoreactive for GFP after intraperitoneal delivery of 10^12^ and 10^13^ vg, and 59.0 ± 2.0% when 10^12^ vg were administered intraductally. This is notably higher than a previous study by Jimenez *et al*., in which single stranded AAV8- or AAV9-CAG-GFP were delivered intraductally and poorly transduced glucagon-secreting cells (approximately 12% of α-cells with 3 × 10^12^ vg)^25^. Xiao *et al*. administered AAV6- and AAV8-CMV-GFP intraductally and transduced approximately 18% and 66% of α-cells, respectively^23^. However, in these studies the authors used the ubiquitous CAG and CMV promoters, whereas here we have employed a fragment of the glucagon promoter to achieve α-cell specificity. It is conceivable that a higher percentage of α-cells could express the transgene if a stronger glucagon promoter fragment were used. Unfortunately, the length of double stranded DNA that can be packaged in an AAV is limited to about 2.2 kb^33^, a fact that notably limits the size of the construct.

In contrast to islets, at 5 months post viral delivery no EGFP immunoreactivity was observed in either intestinal L-cells or in the solitary tract nucleus (NTS) of the brainstem, tissues known to possess proglucagon promoter activity^27,28^. However, we cannot rule out the possibility that EGFP expression occurred at earlier timepoints. Reportedly, whereas 1252 bp of the rat proglucagon 5′ flanking sequences direct expression to α-cells and brain^34^, a longer fragment (2.2 kb) is required for expression in intestinal endocrine cells^35^, which indicates that different DNA sequences are necessary for tissue specific expression. Based on our results, a short fragment (700 bp) of the glucagon promoter is sufficient for expression in islet α-cells.

Obtaining a pure fraction of α-cells is desirable for studies of this cell population; however, it is also very challenging since there is typically considerable contamination with neighboring islet cells^36,37^. Here, we trypsinized and sorted islet cells by FACS, based on the fluorescence emitted by transgenic EGFP^+^ cells, 2–3 months after intraperitoneal AAV GCG-EGFP administration. Whereas 86% of the cells sorted by FACS stained for glucagon, a total of 64% of the sorted cells contained EGFP immunoreactivity. The loss of EGFP signal observed from the immunocytochemical analysis could result from EGFP leaking out of the permeabilized membrane, or lower sensitivity of the immunocytochemical method compared to FACS. Gene expression analysis of the major hormones expressed in the endocrine pancreas revealed prevailing expression of glucagon in the EGFP^+^ fraction, with a small amount of pancreatic polypeptide. Although it is possible that some pancreatic polypeptide-secreting cells (PP cells) were sorted together with α-cells in small doublets that escaped the doublet discrimination strategy, previous studies have shown that a subset of α-cells co-express pancreatic polypeptide^38,39^.

We administered the AAV GCG-EGFP to C57BL/6 adult mice at different doses, up to 10^13^ vg, and observed no differences in body weight, fasting blood glucose, plasma glucagon or insulin levels when compared to the PBS control group. In addition, mice treated with AAV GCG-EGFP displayed normal glucose excursions following a glucose load or an arginine injection, and secreted glucagon appropriately in response to arginine. Therefore, we conclude that the administration of AAV GCG-EGFP at the doses and routes of administration tested does not significantly alter either glucagon or insulin secretion, or glucose homeostasis. Our single cell analysis further suggests that AAV GCG-EGFP does not impair α-cell function. We compared the intracellular Ca^2+^ pattern of α-cells that do not express the transgene (EGFP^−^ α-cells), identified by their responsivity to adrenaline^40,41^, with α-cells that do express the transgene (EGFP^+^ α-cells), and found 5 feature clusters within the entire population that were not driven by cell type. Whereas all α-cells imaged responded to adrenaline, 18.5% and 18.2% of EGFP^−^ and EGFP^+^ cells, respectively, responded to low (0.5 mM) glucose by displaying [Ca^2+^]_i_ oscillations that were later inhibited by high (11 mM) glucose. These results are in agreement with others that also reported a low percentage of single α-cells displaying [Ca^2+^]_i_ oscillations at low glucose concentrations^13,40^, likely due to the loss of cell connections after islet isolation^42^. Some cells (11.3% and 15.2% of EGFP^−^ and EGFP^+^ cells, respectively) exhibited cytosolic Ca^2+^ oscillations in response to both low and high glucose concentrations, similar to what others have observed^14,43^. Even though high glucose is considered a major physiological inhibitor for glucagon secretion, dispersed rodent α–cells display lower responsiveness to glucose inhibition than α-cells within intact islets^14,44^, possibly due to loss of paracrine signals. Molecules released from islet β- and δ-cells under high glucose conditions, including somatostatin, insulin, GABA and Zn^2+^, may inhibit glucagon release from α–cells^45^. Islet isolated α–cells lack such paracrine input and this may explain why the majority of our imaged α-cells that showed Ca^2+^ oscillations at low glucose presented a similar pattern at high glucose. The results we obtained demonstrate that the intracellular Ca^2+^ pattern is intact in EGFP^+^ cells when compared to the non-transgenic α-cells, as has been previously reported for α-cells expressing yellow fluorescent protein (YFP)^13^. Given that EGFP may interfere with Fura-2 signals, other Ca^2+^-indicator dyes such as X-Rhod would be recommended for quantitative analysis^46^.

Taken together, our findings demonstrate that the serotype AAV8 is able to successfully direct the expression of a transgene to approximately half of the α-cell population when administered intraperitoneally at a dose that does not affect glucagon and insulin secretion, or glucose homeostasis. In addition, a single injection of 10^12^ vg of the AAV GCG-EGFP allows α-cells to be visualized based on the endogenous fluorescence emitted by EGFP. This novel method to identify α-cells presents some advantages over previous approaches. For instance, a common strategy to obtain α-cells expressing a fluorescent protein is to cross reporter mice with Gcg-Cre mice^13–16^, requiring added time and cost to breed and genotype mice. The AAV GCG-EGFP allows for the identification of mouse α-cells without the need to cross in a reporter strain, consequently reducing the number of mice that are generated and also allowing for the study of α-cells from different strains, such as various diabetes-prone mouse strains. In addition, with our glucagon promoter we directed the transgene expression to islet α-cells while transgenic models may fail to be as specific, with expression of the transgene detected in other islet cells^47^. In the transgenic GLU-Venus mouse, which expresses yellow fluorescent protein in α-cells, there is also fluorescence in GLP-1 expressing L-cells and brainstem^48^. The AAV GCG-EGFP approach may therefore be preferable when expression in these other sites is not desired. Our approach may also be applicable to other species such as rats; however, this would require confirmation. Finally, our results give proof of principle that potentially therapeutic transgenes, or genes aimed at probing α-cell development or function, could be delivered specifically to α-cells by means of AAVs.

## Material and Methods

### Ethics statement

All procedures with animals were approved by the University of British Columbia Animal Care Committee (animal protocol ID A10-0262) and carried out in accordance with the Canadian Council on Animal Care guidelines.

### Plasmid preparation

The AAV GCG-EGFP plasmid was generated from the dsAAV RIP-EGFP plasmid, kindly provided by Dr. Paul D. Robbins (Department of Microbiology and Molecular Genetics, University of Pittsburgh, Pittsburgh, PA). The rat insulin promoter was excised from dsAAV RIP-EGFP with *BamHI* and *AgeI*. In its place, a BamHI and AgeI-digested amplicon, containing 700 bp of the rat proglucagon promoter and 38 bp of the first exon (UTR), was inserted. The primers used were (5′-3′) CAGCAGGGATCCGTCGACGCTAGCGTAACAGCATGCATCTCAGACCAATAGT (fwd) and CAGCAGACCGGTCTCGAGCTGCAGAGATCTGAGTGTGTTCTGCGCCCAAGCT (rev); underlined bases are proglucagon-specific, with several restriction sites introduced at the 5′ ends of each primer. The recombinant dsAAV rGCG-EGFP plasmid was validated by sequencing and packaged into dsAAVs at the Children’s Hospital of Philadelphia (CHOP; Philadelphia, PA).

### Animals

All experimental C57BL/6 male mice were 6–10 week-old at the time of the AAV administration and were obtained from the Centre for Disease Modeling (Vancouver, Canada) or the Jackson Laboratory (Bar Harbor, ME). All mice employed in this study were housed under a 12 h light, 12 h dark cycle (lights on at 7 AM) at 20–22 °C and had ad libitum access to chow diet (2918, Harlan Laboratories, Madison, WI) and water. *In vivo viral vector administration*. The administration of AAV GCG-EGFP (or sterile PBS for the control group) was performed intraperitoneally or intraductally (via the pancreatic duct), as previously described^22^. Animals recovered for at least 8 weeks before proceeding with any *in vivo* or *in vitro* experiment, except for the fasting blood glucose monitoring in the intraperitoneal study, which was performed weekly from a few days before the AAV/PBS injections until 4.5 months post-injection.

### Glucose and arginine tolerance test

Mice were fasted for 4 h (9 AM–1 PM) and then given either an oral glucose gavage (1.5 g/kg body weight) of a 30% glucose solution or an intraperitoneal injection of 2 g/kg body weight of arginine solution. Tests were performed in the same mice, with the arginine tolerance test performed 3 weeks following the glucose tolerance test. Blood was sampled from the saphenous vein in conscious mice and measured for glucose at 0, 7, 15, 30, and 60 min, and for glucagon at 7 and 60 min after administration of arginine. Blood glucose levels were measured using a One Touch Ultra glucometer (Life Scan Inc., Burnaby, Canada) and plasma glucagon levels were determined by a glucagon RIA kit (Mouse Glucagon RIA Cat. # GL-32K; Millipore). Due to the limited amount of plasma that could be obtained from the animals at the different time points during the arginine tolerance test and assay volume requirements, samples had to be diluted to be assayed for glucagon. Sample dilution was not required for the insulin assay. Blood volumes collected per timepoint were ≤10 µL for glucose, ≤20 µL for insulin, and ≤100 µL for glucagon measurements.

### Fasting glucose, glucagon and insulin levels

Ten weeks after the arginine tolerance test, mice were fasted for 4 h (9 AM–1 PM) for blood glucose measurements, overnight (8 PM–9 AM) for plasma insulin and glucagon analysis, and fed *ad libitum* for 2 h after an overnight fast for plasma glucagon measurements in the re-fed state. Blood was collected from the saphenous vein for subsequent measurements. Blood glucose and plasma glucagon levels were measured as described above. Plasma insulin levels were determined by an Insulin Mouse Ultrasensitive enzyme-linked immunosorbent assay (ELISA) (Cat. # 80-INSMSU-E01 ALPCO Diagnostics, Salem, NH).

### AST and ALT activity measurements

Serum AST and ALT activities were measured by using commercially available kits (Sigma-Aldrich, St. Louis, MO).

### Islet isolation, dispersion and cell culture

For experiments performed with dispersed islet cells (FACS and Ca^2+^ recordings), mice were sacrificed by CO_2_ prior to islet isolation. These mice had not been previously employed for any metabolic test related to glucose homeostasis. Hank’s balanced salt solution (HBSS) containing (in mM): NaCl, 137; KCl, 5.4; NaH_2_PO_4_, 4.2; KH_2_PO_4_, 4.1; HEPES, 10; MgCl_2_, 1; glucose, 5; and 1000 U/mL of type XI collagenase (Sigma-Aldrich, St. Louis, MO) was used to isolate pancreatic islets. Pancreata were injected via the pancreatic duct with the collagenase-containing solution prior to their removal and digested at 37 °C for 12 min. Islets were then washed with iced-cold HBSS and handpicked under a microscope. Freshly isolated islets were dispersed into single cells by trypsin enzymatic digestion, and cultured in RPMI 1640 (Sigma-Aldrich, St. Louis, MO) containing 11.1 mM glucose, 10% FBS, 100 IU/mL penicillin, 0.1 mg/mL streptomycin (2%), and 1% Glutamax 100X.

### Ca^2+^ imaging and cluster analysis

Single pancreatic cells were imaged following 24 h culture in RPMI 1640 (Sigma-Aldrich, St. Louis, MO) containing 11.1 mM glucose, 10% FBS, 100 IU/mL penicillin, 0.1 mg/mL streptomycin (2%), and 1% Glutamax 100X, at 37 °C and 5% CO_2_ on glass coverslips, on a Zeiss Axiovert 200 M inverted microscope equipped with a temperature-controlled stage and a FLUAR 20X objective (Carl Zeiss, Thornwood, NY). Cells were loaded with 2.5 μM Fura 2-AM for 30 min in the culture medium and continuously perifused with Ringer’s solution containing (in mM): NaCl, 144; KCl, 5.5; CaCl_2_, 2; MgCl_2_, 1; HEPES, 20 (adjusted to pH 7.35 by NaOH) during the experiments, at either 0.5 mM or 11 mM glucose. The wavelengths of fluorescent excitation were controlled by means of excitation filters (Chroma Technology, Rockingham, VT) mounted in a Lambda DG-4 wavelength switcher (Sutter Instrument Company, Novato, CA). Fura-2 AM was excited at 340 nm and 380 nm, and the emitted fluorescence was monitored through a D510/80 m filter. Changes in [Ca^2+^]_i_ were expressed as the ratio of the fluorescence emission intensities. Images were acquired using a CoolSNAP HQ digital camera (Roper Scientific, Tucson, AZ). Slidebook software package (Intelligent Imaging Innovations, Inc. Denver, CO) was employed for image acquisition and imaging analysis.

Taking a similar approach to that described in Wills *et al*.^30^, 8 features were extracted from the Ca^2+^ traces. Response to glucose or adrenaline were defined as the median high glucose (11 mM) or adrenaline (5 µM) signal above baseline (0.5 mM), respectively, normalized to the maximum response to adrenaline above baseline. High glucose oscillation, low glucose oscillation, and adrenaline oscillation were defined as the median absolute deviation (MAD) during the high glucose, low glucose or adrenaline exposures, respectively, normalized to the maximum response to adrenaline above baseline. Finally, peaks during each phase were identified as local maxima reaching a value with a percent difference above the median baseline level greater than 20%. Using the mclust package^49^ in R^50^, the best fitting model was identified using Bayesian Information Criterion. Figures were generated using the ggplot2 package^51^. Code used to analyze data and generate figures are available upon request.

### Tissue fixation and immunostaining

The mice employed for the *in vivo* procedures were sacrificed 5 months post intraperitoneal AAV-delivery, and other mice were sacrificed 2 months post intraductal AAV-administration for immunohistochemistry analysis. Pancreas, liver, gut and brain were harvested and fixed in 4% paraformaldehyde (PFA) overnight, after transcardial perfusion with PBS for 2 min and ice-cold 4% PFA for 10 min in mice placed under isoflurane anesthesia. After fixation, the small intestine, liver and pancreata were washed in ethanol (EtOH) 70% and embedded in paraffin. Brains were left in PBS containing 25% sucrose for 4 days and then frozen in −40 °C 2-methylbutane for subsequent sectioning of the brainstem on a sliding microtome into 30 µm coronal sections. Brainstem sections were stored in cryoprotectant at −20 °C prior to immunohistochemistry for GFP and GLP-1. For immunocytochemistry of islet cells, dispersed cells that were sorted by FACS were cytospinned for 3 min at 1500 rpm. Then, cells were fixed with 4% PFA for 10 min at room temperature, dehydrated for 3 min in EtOH 30%, 50% and 70%, and permeabilized with 0.5% Triton X-100. Primary antibodies against glucagon (mouse, 1:1000, Sigma-Aldrich Cat. #G2654, St Louis, MO), GFP (rabbit, 1:500; Life Technologies Cat. #A11122), GLP-1 (mouse, 1:100000; kindly provided by Prof. David A. D′Alessio, Department of Medicine, Duke University School of Medicine, Durham, NC), and insulin (guinea pig, 1:1000; Linco Cat. #4011-10 F) were used for an overnight incubation at 4 °C. For secondary antibodies, goat anti-mouse Alexa 594 (1:500; Invitrogen Cat. #A11032), goat anti-rabbit Alexa 488 (1:500 Cat. #A11034; Invitrogen) or donkey anti-rabbit Alexa 488 (1:500; Invitrogen Cat. #A21206) were added and incubated for 1 hour at room temperature. For immunohistochemistry in the brainstem, every 6^th^ section from the midbrain to the caudal medulla was incubated with a mouse GFP antibody (1:100; Cat. #MAB3580, Millipore) and a rabbit GLP-1 antibody (1:500, Cat. #Y320, Yanaihara Institute) at 4 °C for 2 days. Following 1 hr secondary antibody incubation (1:200, goat anti-rabbit 594 and goat anti-mouse 488; Invitrogen), floating sections were mounted onto slides for imaging.

### Fluorescence activated cell sorting

Immediately after dispersion with trypsin/EDTA, islet cells were washed in cold Ca^2+^-free PBS, carefully spun at 1000 rpm and resuspended in 10% FBS/Ca^2+^-free PBS for FACS analysis. Sorting was performed on a BD Ilu Aria using an 85-μm nozzle at 25 psi. The excitation laser was 488 nm, and the emission filter was 530/30 for the FITC parameter and 610/20 for the PE-Texas Red parameter.

### RNA isolation and qPCR

Immediately after cell sorting, total RNA was extracted with Trizol (Invitrogen) reagent according to the manufacturer’s instructions and quantified with a Nanodrop 1000 (Thermo Scientific), followed by cDNA synthesis (iScript cDNA synthesis kit; Bio-Rad). Quantitative RT-PCR (qRT-PCR) was performed using SYBR Green master mix (Exiqon) with the following primer sequences (5′-3′): CAGGCACGCTGATGGCT and GTGAAGATGGTTGTGAATGGTGAAATA (*Gcg*, NM_008100.4), TGATCCGCTACAATCAAAAACCAT and CCACCTCCAGTGCCAAG (*Ins*, NM_008387.5), CTGAGCAGGACGAGATGA and TAAGAGGATGTGAATGTCTTCCAGAA (*Sst*, NM_009215.1), CGCATACTGCTGCCTCTCC and CCTGGTCAGTGTGTTGATGTATCTG (*Ppy*, NM_002722.4), and GCAGATTGTTTGGAATGGTC and TGCTCACATGGCTGACTTTA for the housekeeping gene (*Pgk1*, NM_008828.3). Relative values were calculated with the delta-delta CT method.

### Sample size

Studies of the *in vivo* effects of AAV-GCG-EGFP and α-cell function by imaging intracellular Ca^2+^ were designed as groups of n = 5. However, for the 5 month *in vivo* follow-up after AAV injection, 3 mice died (one mouse from the PBS group, one mouse from the 10^10^ vg group, and one mouse from the 10^13^ vg group), therefore reducing these groups to n = 4. In addition, some plasma and α-cell preparations were too small for analysis. For studies where our aim was to confirm whether a pure population of glucagon-secreting α-cells could be sorted, we used 3–5 mice per group.

### Statistical analysis

Data are expressed as mean ± SEM. Statistical analysis was performed using Kruskal-Wallis test or two-way ANOVA followed by Bonferroni test (GraphPad Prism, GraphPad Software Inc., La Jolla, CA, USA). Correlations between categorical variables were performed using a Chi-squared test with Monte Carlo simulation to calculate an exact P value and Cramer’s V for effect size^50^. P values ≤ 0.05 were considered significant.

## Supplementary information

Supplementary Figures
