# Supplementary material for: AAV GCG-EGFP, a new tool to identify glucagon-secreting α-cells
Source: Sci Rep. 2019 Jul 25;9:10829. doi: 10.1038/s41598-019-46735-2 (PMC6658537; doi:10.1038/s41598-019-46735-2)
Supplement: Supplementary file 1 — Supplementary Figures [file 41598_2019_46735_MOESM1_ESM.pdf]

AAV GCG-EGFP, a new tool to identify glucagon-secreting α-cells

Eva Tuduri, Maria M. Glavas, Ali Asadi, Robert K. Baker, Cara E. Ellis, Galina Soukhatcheva, Marjolaine Philit, Frank K. Huynh, James D. Johnson, C. Bruce Verchere, Timothy J. Kieffer

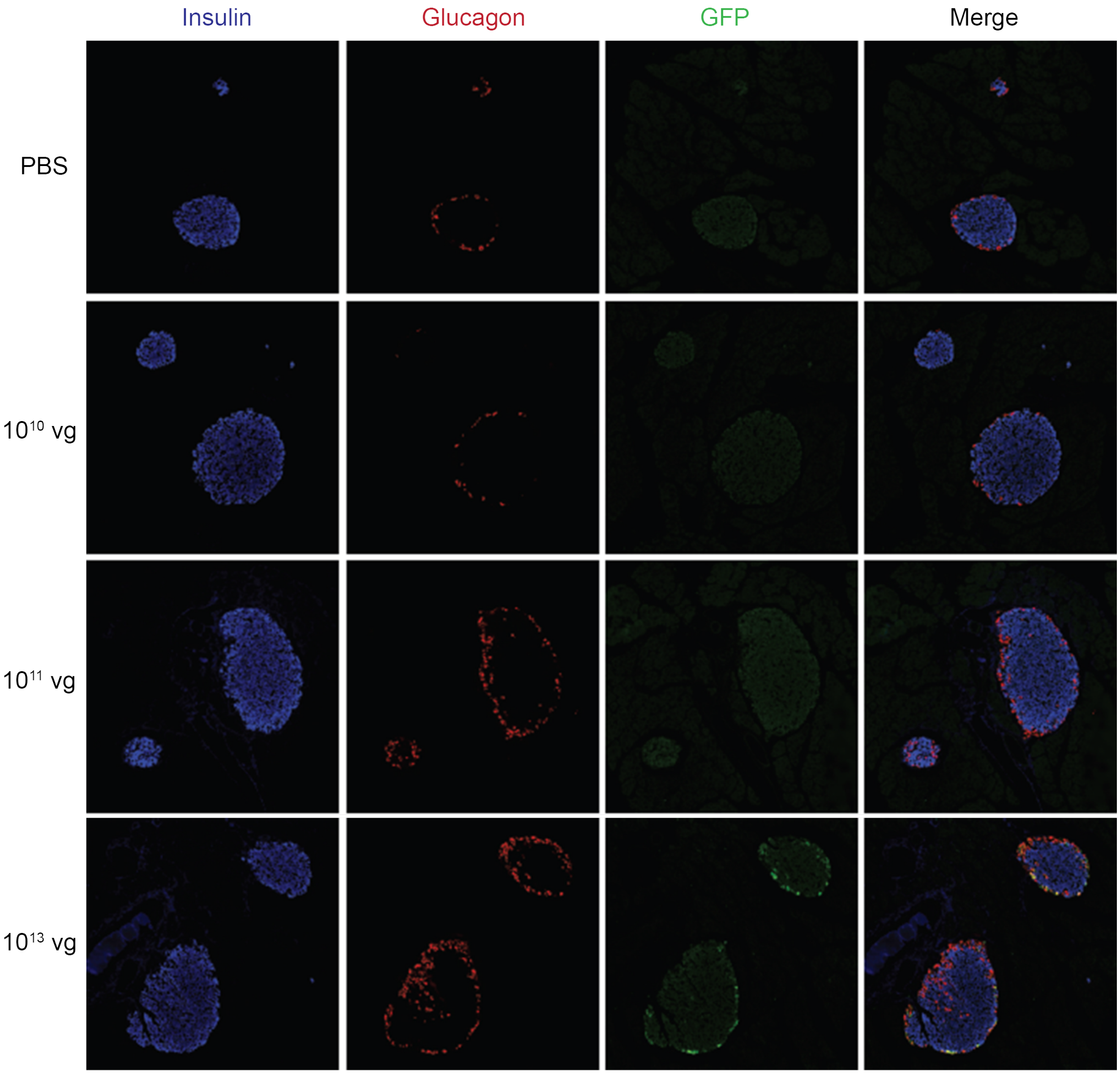

Supplementary Figure S1. Low doses of AAV GCG-EGFP do not lead to α-cell EGFP expression. Pancreas sections from adult C57BL/6 mice treated with AAV GCG-EGFP by single intraperitoneal injection of PBS, low doses (10^{10} and 10^{11} vg) and high dose (10^{13} vg) of AAV GCG-EGFP. Insulin (blue), glucagon (red) and GFP (green).

GFP

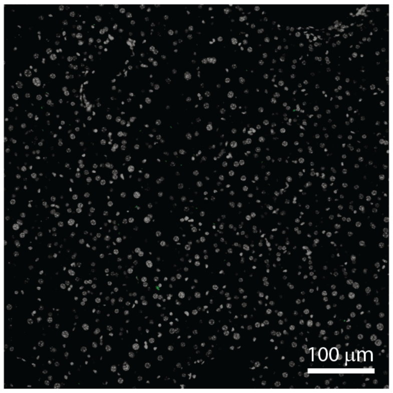

Supplementary Figure S2. Representative liver section from adult C57BL/6 mouse treated with a single intraperitoneal injection of 10^{13} vg (high dose) AAV GCG-EGFP. No GFP (green) staining was observed with the exception of autofluorescence in the occasional blood cell. Nuclei (DAPI) are labelled in grey. Scale bar = 100 µm.

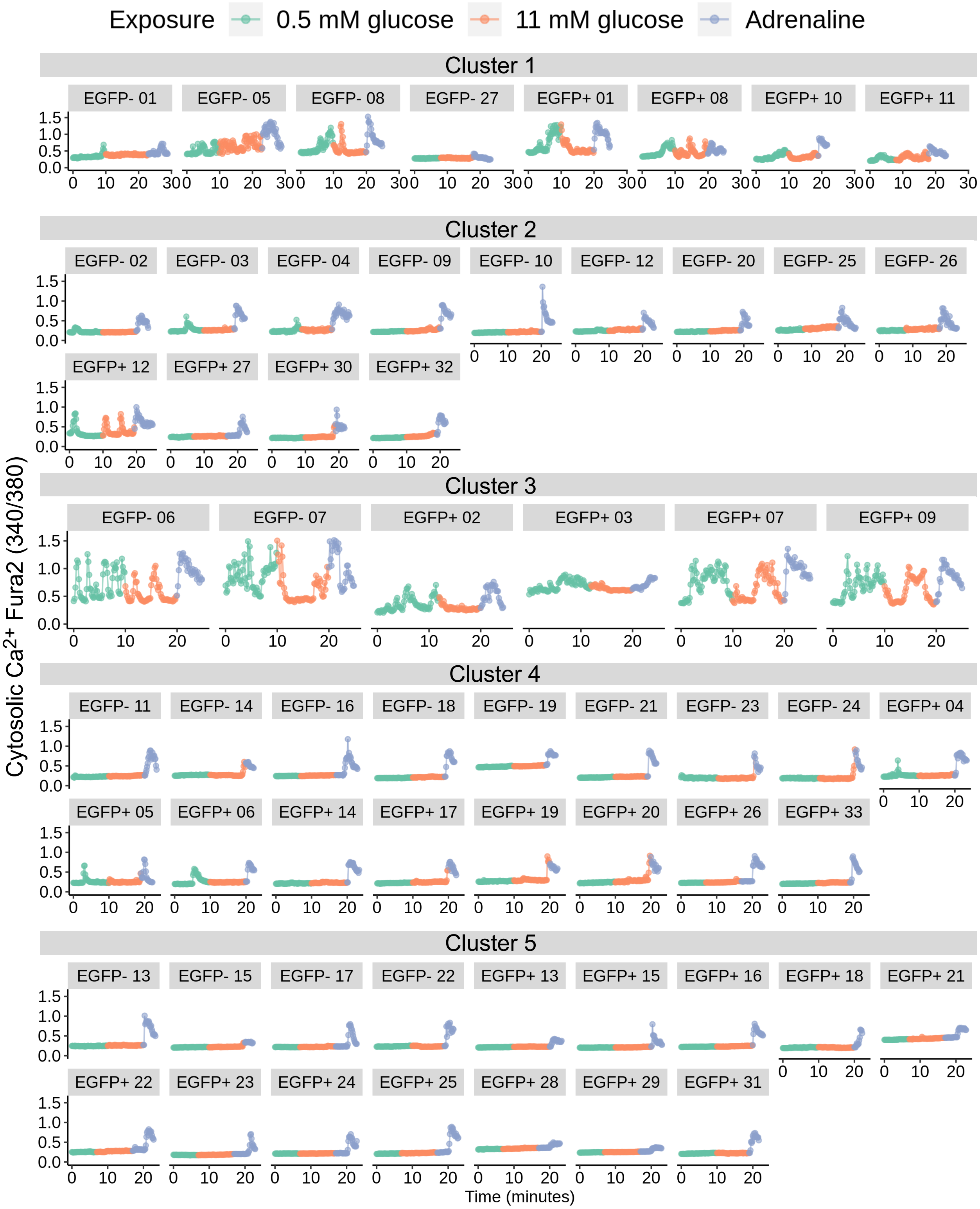

Supplementary Figure S3. Individual Ca^{2+} traces of $EGFP^{+}$ and $EGFP^{-}$ α-cells, in response to low glucose (0.5 mM), high glucose (11 mM) and adrenaline (5 uM). A total of 33 $EGFP^{+}$ and 27 $EGFP^{-}$ α-cells from 5 AAV-treated mice were imaged and grouped into 5 feature clusters based on responsivity to the different stimuli. Trace labels refer to individual cell number and $EGFP^{-}$ or $EGFP^{+}$ status.
